# Molecular Imaging Probes for Diagnosis and Therapy Evaluation of Breast Cancer

**DOI:** 10.1155/2013/230487

**Published:** 2013-02-26

**Authors:** Qingqing Meng, Zheng Li

**Affiliations:** Department of Translational Imaging, The Methodist Hospital Research Institute, Weill Cornell Medical College, 6670 Bertner Avenue, Houston, TX 77030, USA

## Abstract

Breast cancer is a major cause of cancer death in women where early detection and accurate assessment of therapy response can improve clinical outcomes. Molecular imaging, which includes PET, SPECT, MRI, and optical modalities, provides noninvasive means of detecting biological processes and molecular events *in vivo.* Molecular imaging has the potential to enhance our understanding of breast cancer biology and effects of drug action during both preclinical and clinical phases of drug development. This has led to the identification of many molecular imaging probes for key processes in breast cancer. Hormone receptors, growth factor receptor, and angiogenic factors, such as ER, PR, HER2, and VEGFR, have been adopted as imaging targets to detect and stage the breast cancer and to monitor the treatment efficacy. Receptor imaging probes are usually composed of targeting moiety attached to a signaling component such as a radionuclide that can be detected using dedicated instruments. Current molecular imaging probes involved in breast cancer diagnosis and therapy evaluation are reviewed, and future of molecular imaging for the preclinical and clinical is explained.

## 1. Introduction 

Breast cancer is a major cause of mortality in women worldwide. In the US, approximately 40,000 women die of breast cancer every year and about 1 in 8 women will be diagnosed with breast cancer over the course of her lifetime. Although mammography remains a key imaging method for screening of breast cancer, the overall accuracy of this test is low [1, 2], particularly in the setting of fibrocystic breast disease and dense breast tissue in young women. There remains a great demand for the ability to define the extent of disease, to monitor treatment response and to predict tumor behavior in breast cancer patients in which molecular imaging may play an important role. Molecular imaging, including positron emission tomography (PET), single-photon emission computed tomography (SPECT), magnetic resonance imaging (MRI), optical imaging, and ultrasound, provides noninvasive *in vivo* information on important biological and molecular events, which can ultimately lead to improved early detection and characterization of therapy response.

The goal of molecular imaging is to detect and quantify biological processes at the cellular and subcellular levels in living subjects. Molecular changes in tissue and organ from functional molecular imaging can be used for comparing to traditional imaging which usually gives only anatomic information. With advancements in instrumentation and introduction of novel targeted probes, molecular imaging firmly establishes its role in drug development and in clinical assessment. The techniques used include scintigraphic modalities (PET/SPECT), magnetic resonance and spectroscopy, optical and fluorescence imaging, and ultrasound. The use of multimodality techniques such as PET-CT and PET-MRI allows the detection of molecular, pathophysiological, and anatomic changes in a single scan.

PET involves administration of radioactive probes and detection of (annihilation) photons produced in the process of radioactive decay and interaction with surrounding tissues. It is an imaging technique that allows the reconstruction of three-dimensional images of functional processes in living subjects. PET was introduced by David E. Kuhl and Roy Edwards from the University of Pennsylvania in the late 1950s. PET emerged as the modality of choice in the clinical setting due to its high sensitivity, good spatial resolution, and proven quantification abilities [3]. Fluorodeoxyglucose (^18^F-FDG) is the most common radiotracer used for PET imaging as it reveals specific tissue metabolic activity and has been used for primary tumor detection and diagnosis, staging of local, regional, and distant metastases, and for monitoring therapy response.

Compared with PET, single photon emission tomography (SPECT) has the advantage of a wider variety of radiopharmaceuticals and overall lower costs but has the disadvantage of limited spatial resolution. Typical radiopharmaceuticals used in SPECT for breast cancer imaging include ^99m^Tc-diphosphonates, [^201^Tl] thallium chloride, ^99m^Tc-tetrofosmin, and ^99m^Tc-methoxyisobutylisonitrile (^99m^Tc-MIBI; ^99m^Tc-sestamibi) [2]. Gamma cameras equipped with multiple detectors can acquire 2D images as well as 3D images (SPECT). MRI has the advantages of high spatial resolution and provides the best soft tissue resolving power of all the imaging modalities, especially when combined with appropriate imaging contrast agents [4]. After more than 10 years of clinical use, breast MRI has become accepted as a complementary technique to radiographic mammography and ultrasound. Breast MRI is frequently used in the management of breast cancer, especially to determine the extent of disease in the breast and to direct local therapy.

Optical imaging includes fluorescence and bioluminescence-based modalities. Charge coupled device (CCD) cameras are used to detect and analyze signal originating from fluorescent and bioluminescent probes. In some applications, further postprocessing of optical images allow for a limited form of 3D rendering. The clinical application of fluorescence and bioluminescence-based optical imaging has been limited mainly due to poor light penetration through body tissues and fluids.

Ultrasound is a low-cost imaging modality which is widely used in both clinical and preclinical settings. The imaging sensitivity and resolution of ultrasound can be enhanced with the administration of microbubble contrast agents [5].

**Figure 1:**
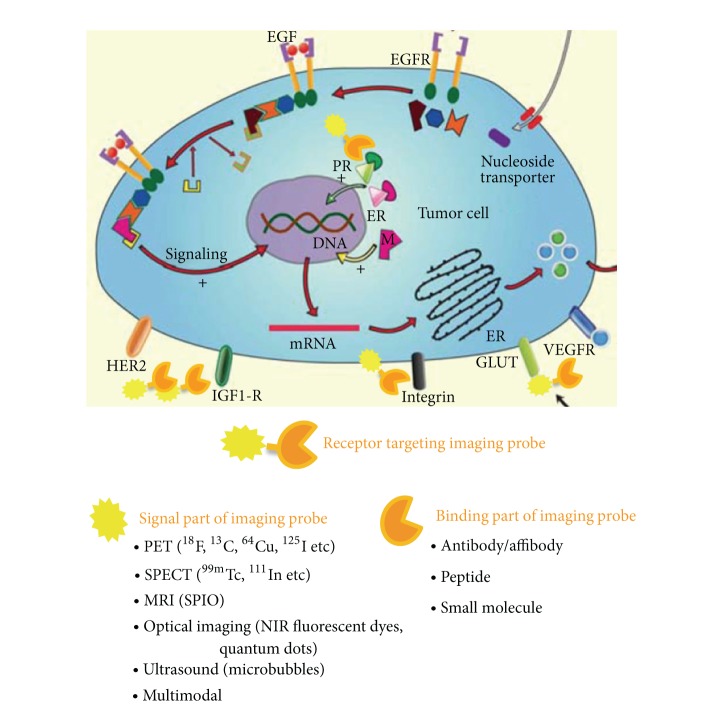
Receptor targeting imaging of breast cancer. Adapted from [84].

**Figure 2:**
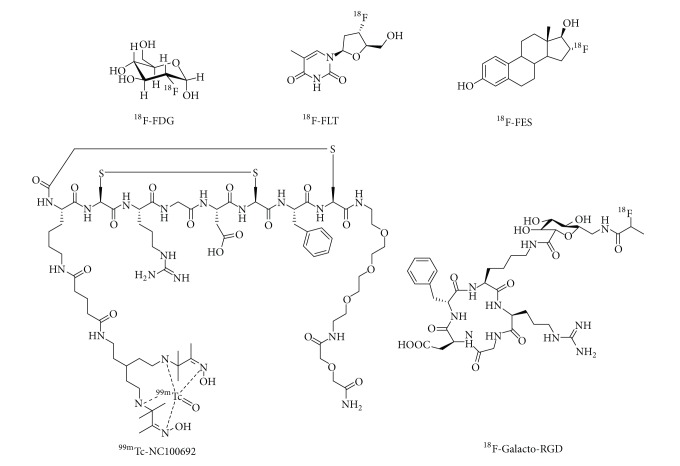
Structures of representative molecular imaging probes in preclinical and clinical trials for breast cancer imaging.

As a key component of molecular imaging, a probe must specifically reach the target of interest *in vivo* and be detectable within a defined span of time. In addition to a target-specific affinity component, molecular imaging probes also include a signaling component that is useful for different imaging modalities shown in Figure 1. Development of a desirable molecular imaging probe with clinical translation potential is frequently a challenging endeavor. Nowadays, the understanding of the breast cancer molecular biology allowed researchers to select suitable targets to develop breast tumor specific probes to enhance our understanding of molecular mechanisms and drug activity during preclinical and clinical drug development. For example, hormone receptors (ER and PR), growth factor receptor (HER2, EGFR, and IGF-1R), and angiogenic factors (VEGFR, integrin *α*_*v*_ *β*_3_) have been adopted as imaging targets to detect/stage the breast cancer and monitor treatment efficacy. This paper mainly summarizes reported antibody, peptide, and small molecule-based molecular targeting probes for PET, SPECT, MRI, and optical imaging for breast cancer diagnosis and therapy evaluation. Molecular imaging probes reviewed were listed in Table 1. Structures of representative breast cancer targeting probes in clinic and clinical trials were shown in Figure 2. There are hundreds of molecular imaging probes reported for breast cancer diagnosis and therapy evaluation [6], but only a few of them (mainly PET tracers) have entered the clinical setting [7, 8].

## 2. Molecular Probes for Imaging Breast Cancer Glucose Metabolism and DNA Synthesis

### 2.1. FDG-PET

FDG-PET has been evaluated for primary breast cancer detection and diagnosis and locoregional and distant sites staging, as well as monitoring therapy response. After being transported across the cell membrane by glucose transporters, Glut-1 and Glut-3, FDG is converted to FDG-6-phosphate under the action of hexokinase. Due to the lack of a hydroxyl group at the 2-position, FDG cannot be further metabolized which leads to its intracellular accumulation within metabolically active tissue such as most solid tumors [83]. Heterogeneity of the disease and tumor size influence the results of FDG-PET for the initial detection and diagnosis of primary breast cancer. FDG-PET still cannot serve as a “metabolic biopsy” as a method of screening for breast cancer. Therefore, FDG with positron emission mammography (PEM) has been introduced as an alternative. PEM has a much higher spatial resolution than whole body PET because it has two opposite detector heads on each side of the breast, which minimizes the distance between the radiation source and the detectors. Schilling et al. have reported that PEM can detect tumor as small as 1.5 mm in diameter with less breast compression and was not affected by breast density [9]. Riegger et al. reported that full-dose, intravenous contrast-enhanced FDG PET/CT was more accurate than conventional imaging for initial breast cancer staging due to the higher detection rate of metastases and synchronous tumors [10]. Dual time point FDG-PET/CT improves the discrimination between non-invasive and invasive tumors and provided superior sensitivity for the detection of small tumors and within dense breasts [11]. Park et al. reported that the combined use of diffusion-weighted MRI and FDG PET/CT has the potential to improve specificity in predicting pathological complete response to neoadjuvant chemotherapy in breast cancer patients [12]. A study showed that FDG-PET/CT plays an important role in staging patients with locoregional breast cancer recurrence [13]. There are also a large number of studies that have used FDG-PET to evaluate breast cancer treatment response [14, 15]. The decrease in the ratio of FDG tumor metabolism to blood flow suggests tumors shift to more aerobic metabolism after chemotherapy. The patients with high FDG uptake are more likely to have poor response and early relapse [16]. However, despite these impressive features, FDG-PET is a relatively nonspecific tracer. Malignancy, acute and chronic inflammation, physiologic lactation, and benign breast masses may show false-positive FDG uptake on PET due to high glucose metabolism. PET imaging probes specifically targeting breast cancer cells are still in high demand.

### 2.2. FLT-PET

[^18^F]fluorothymidine (FLT) is a pyrimidine analogue which was introduced for tumor imaging by Grierson and Shields in 1998 [17]. Phosphorylated by S-phase-specific thymidine kinase 1, FLT is trapped intracellularly by entering the salvage pathway of DNA synthesis without incorporation into DNA [18]. FLT-PET detects cellular proliferation which is believed to be more specific for tumor tissue than FDG-PET. In a pilot study, 12 patients with 14 primary breast cancer lesions (T2–T4) were studied by FLT-PET [19]. Compared with FDG-PET, the SUVs of primary tumors (5/6) and axillaries lymph node metastases (3/4) were lower in FLT-PET. However, FLT uptake in surrounding breast tissue was also lower which caused the tumor contrast to be comparable to that with FDG. The result indicated that FLT-PET was suitable for the diagnosis of primary breast cancer and locoregional metastases. FLT-PET has been studied for evaluating therapy response in breast cancer patients [20]. A significant decrease in FLT uptake was found after docetaxel treatment. Changes in tumor proliferation assessed by FLT-PET predicted the therapy response after initiating docetaxel, which gave the chance to stop therapy in the case of non-FLT-PET response. Kenny et al. assessed the altered pharmacokinetics of FLT in patients following administration of capecitabine, a thymidylate synthase (TS) inhibitor [21, 22]. In this clinical imaging study, FLT uptake in patients was increased in tumors but not in normal tissue within 1 hour following treatment with capecitabine with implications for use of FLT-PET in imaging TS inhibition in breast cancer patients. Although FLT-PET is not regarded as a routine staging tool for breast cancer, it is a promising tool for the prediction of therapy response.

## 3. Molecular Probes for Imaging Breast Cancer Specific Targets

### 3.1. Imaging Probes Targeting Hormone Receptors

The hormones (progesterone and estrogen) play a critical role in the initiation and progression of breast cancer. There are 4 subtypes of breast cancer: (1) luminal A, ER+ but low grade, (2) luminal B, ER+ but high grade, (3) HER2+ type, and (4) triple negative (ER-, PR-, and HER2-) [85]. The majority of breast cancers have high expression of estrogen receptors (ER) and progesterone receptor (PR).

#### 3.1.1. ER

ER is a ligand-dependent transcription factor and is activated by estradiol, an endogenous estrogen, and subsequently regulates several downstream target genes [86]. ER contains two subtypes, ER*α* and ER*β*, which interact with the same genes. ER*α* and ER*β* have different expression patterns and levels which normally determine their functional outcomes. ER*α* is the dominant receptor in breast cancer cells. Imaging probes based on estradiol derivatives and related endocrine drugs were reported for ER targeting in breast cancer.

*Estradiol-Based ER Imaging Probes.* The ^18^F, ^123^I, and ^99m^Tc labeled estradiol derivatives have been developed and tested for the assessment of ER expression in breast cancer. [^18^F]fluoroestradiol (FES) binds to both subtypes ER*α* and ER*β*, with a preference for ER*α* [87]. Linden et al. used FES-PET imaging to evaluate hormonal therapy response in metastatic breast cancer patients [23]. They showed that FES uptake in PET imaging was correlated with ER expression assayed by qualitative immunohistochemistry measurement. This study suggested that quantitative FES-PET was useful to predict treatment effect of salvage hormonal therapy and to guide breast cancer therapy selection. Another estradiol analog probe, 4,16*α*-[16*α*-^18^F]difluoro-11*β*-methoxyestradiol (4F-M[^18^F]FES), was also developed as a PET tracer for the studies of the ER status in primary and metastatic breast cancer [24, 25]. Iodine-123-labelled cis-11*β*-methoxy-17*α*-iodovinyloestradiol (Z-[^123^I]MIVE) was reported for gamma imaging of estrogen receptors (ERs) in human breast cancer [26, 27]. The potential of both MIVE stereoisomers (E- and Z-[^123^I]MIVE) was studied. Both isomers of MIVE showed high affinity *in vitro* and *in vivo;* however, the binding affinity of Z-MIVE was manyfold higher than that of E-MIVE. In addition, increased focal uptake at known tumor sites was found in planar whole body imaging of two breast cancer patients 1-2 hr after injection of Z-[^123^I]MIVE. Preclinical studies using ^99m^Tc(I)-estradiol-pyridin-2-yl hydrazine derivatives and ^99m^Tc-glutamate peptide estradiol (GAP-EDL) were reported for functional SPECT imaging of ER-positive breast tumors [28, 29].

*Endocrine Drugs-Based ER Imaging Probes*. ^18^F and ^99m^Tc radiolabeled endocrine drugs were investigated for the imaging of ER expression. ^18^F radiolabel tamoxifen (FTX) was first obtained by Yang and colleagues for the imaging of mammary tumors in rat models [30]. Two years later, the clinical study of FTX was reported in 10 patients of 23 ER-positive suspected primary or metastatic lesions [31]. The study demonstrated that FTX PET imaging is useful to predict tamoxifen therapy response. As reported, the tumors with good drug response had higher average SUVs than those with poor response (2.46–0.62 versus 1.37–0.59, *P* < 0.05).

F-18 radiolabeled cyclofenil analogues were investigated for imaging of ER-positive breast tumors with PET [32, 33]. C3 site of cyclofenil analogues is more tolerant of steric bulk and polar groups than the C4 site according to the binding affinity to both ER*α* and ER*β*. Toremifene (TOR), a chlorinated analog of tamoxifen, was coupled with diethylenetriamine pentaacetic acid (DTPA) and then radiolabeled with ^99m^Tc to form ^99m^Tc-DTPA-TOR [34]. The SPECT tracer exhibited high breast tissue/background ratio in xenograft tumors. Gao et al. developed the carbon-11-labeled tetrahydroisoquinoline-derivatives as radioligands for PET imaging of ER expression in breast cancer [35]. Compared with 4-hydroxytamoxifen, the tetrahydroisoquinoline based probes displayed similar imaging ability in MCF-7 cell lines *in vitro*.

To permit selective noninvasive imaging of ER-positive tumors *in vivo, *an MRI probe based on pyridine-tetra-acetate-Gd (III) chelate (PTA-Gd) was developed [4]. PTA-Gd conjugated to 17*β*-estradiol (EPTA-Gd) or to tamoxifen (TPTA-Gd) was examined in ER-positive or ER-negative tumors. *In vivo* competition experiments confirmed that the enhanced detection capability of EPTA-Gd was based specifically on ER targeting that could differentiate ER-positive and ER-negative tumors. Unfortunately, TPTA-Gd accumulated selectively in muscle and could not preferentially identify ER-positive tumors.

#### 3.1.2. PR

Progesterone receptor (PR) is crucial for the growth of breast cancer, and its level is regulated by ER. PR level is used in the diagnosis and to predict the success of anti-estrogen treatment in breast cancer. Compared with ER imaging, there is limited progress in clinical PR imaging which might facilitate therapeutic advancement as well as breast tumor diagnosis.

21-[^18^F]fluoro-16-*α*-methyl-19-norprogesterone ([^18^F]FMNP), a steroidal progestins, showed that tissue uptake correlates well with progesterone receptor expression, which demonstrates its potential applicability for imaging PR-positive tumors by PET [36]. ^18^F-labeled steroidal progestin, 21-[^18^F]-fluoro-16*α*-ethyl-norprogesterone ([^18^F]FENP), was found to have selective high binding affinity in target tissues of estrogen-primed rats but was not a suitable agent for imaging progestin receptors in humans [37].

Tanaproget is a potent nonsteroidal PR agonist with very high binding affinity and excellent *in vivo* activity [38, 39]. A series of fluoroalkyl-substituted 6-aryl-1,4-dihydrobenzo [d][1,3]oxazine-2-thiones, analogues of Tanaproget, have been evaluated as potential PET imaging agents for breast cancer diagnosis. 4-[^18^F]Fluoropropyl-Tanaproget ([^18^F]FPTP) was prepared and evaluated for imaging PR levels by PET. The biodistribution of [^18^F]-FPTP is comparable to that of F-18-labeled steroidal progestins, FENP, and FFNP. [^18^F]-FPTP exhibited high target tissue uptake efficiency and selectivity, as well as prolonged retention. The results showed that [^18^F]-FPTP could be a PET imaging probe for PR-positive breast tumors.

### 3.2. Imaging Probes Targeting Growth Factor Receptors

#### 3.2.1. HER2

Human epidermal growth factor receptor 2 (HER2), as well as HER1 (EGFR, ErbB1) and HER4, belongs to the epidermal growth factor receptor (EGFR) family [88]. EGFR members are involved in the regulation of cell growth, differentiation, and survival [89]. The overexpression of HER2 is found in many kinds of tumor cells including breast, ovarian, bladder, prostate, colon, stomach, kidney, and nonsmall lung cancer cells [90–93]. Overexpression of HER2 occurs in 25% to 30% of all breast cancers, and it is strongly associated with increased disease recurrence and a worse prognosis. Trastuzumab (Herceptin), an antibody binds selectively to HER2, is in clinic for HER2 positive breast cancer patients [94–96]. Molecular imaging of HER2 has been a useful tool to assess HER2 expression and to monitor therapy response.

*Antibody Based-HER2 Imaging Probes*. Antibody trastuzumab- or pertuzumab-based imaging probe has been developed by several groups for *in vivo* imaging of HER2 [97]. ^89^Zr-labeled trastuzumab and ^111^In-labeled trastuzumab were developed to detect HER2 positive lesions in patients with metastatic breast cancer [40, 41]. ^111^In-labeled pertuzumab was used to study the inhibition of HER2 in human breast cancer xenografts with trastuzumab treatment [42]. This study indicated that early assessment which leads to the prediction of the efficacy of therapy can be realized by monitoring the level of HER2 by SPECT imaging. 2Rs15d, a small HER2-binding fragment derived from heavy-chain-only antibodies, was developed as HER2 SPECT probe [43]. The results showed that ^99m^Tc-labeled 2Rs15d had high HER2-specific binding affinity and tumor uptake in two HER2-positive tumor models. Fast blood clearance, low accumulation in nontarget organs except kidneys, and high tumor-to-blood and tumor-to-muscle ratios were observed in mouse models at 1 hour after injection.

For MRI, dextran-modified iron oxide nanoparticles was conjugated to trastuzumab to provide a HER2-specific MR probe which could detect low HER2 expression in cell lines *in vitro* [44]. Yang et al. reported the poly(amino acid) coated iron oxide nanoparticles conjugated with HER2 antibody. The resulting tracer detected breast cancer cells and enhanced signal intensities in T(2)-weighted images [45]. A multimodal method combined with SPECT and optical imaging was reported for the detection of HER2 expression using ^111^In and indocyanine green (ICG) dual labeled panitumumab (anti-HER1) and trastuzumab [46]. This multifunctional probe made it possible to measure the level of HER2 by optical imaging and SPECT simultaneously.

*Affibody-Based HER2 Imaging Probes*. To improve the low tumor penetration and slow clearance caused by the large size of a full antibody, affibody was developed and used as a HER2-specific ligand [98, 99]. Affibody is stable and hydrophilic and its small size leads to rapid blood clearance and good tumor penetration without losing high binding affinity to HER2. DOTA-functionalized affibody ABY-002 (HER2:342-pep2) was labeled with ^68^Ga to image HER2-positive tumors by PET [47]. The study demonstrated that ^68^Ga-ABY-002 was rapidly cleared from blood and tissue (except kidneys) with high tumor uptake at 2 hrs after injection in a mouse model. For SPECT imaging, Ahlgren et al. reported an affibody-based tracer ^99m^Tc-Z_HER2:2395_-Cys which showed visualization of HER2-expressing tumors [48]. For MRI, a combination of biotinylated HER2-specific affibody and streptavidin-funtionalized superparamagnetic iron oxide (SPIO) was reported to successfully image HER2-positive tumors [49]. By using affibody-based fluorescence agent, optical imaging was also applied in quantitatively monitoring tumor HER2 expression *in vivo *[50, 51].

*Treatment Response Evaluation*. Besides monitoring the level of HER2 expression in tumor, HER2 imaging has been used for assessment of HER2 downregulation in response to anti-Hsp90 therapy. Smith-Jones et al. reported PET imaging of ^68^Ga-labeled F(ab′)2 fragments of Herceptin to determine the kinetics of loss and recovery of HER2 expression in response to the Hsp90 inhibitor 17-AAG in BT-474 human breast cancer xenografts [52]. This study showed that HER2 expression level estimated by PET imaging declined 50% 24 hrs after drug administration and remained fairly constant over the next 5 days. By contrast, the control group had a 20% increase in HER2 expression over the same 7-day period. Oude Munnink et al. used ^89^Zr-labeled trastuzumab to evaluate HER2 expression changes following treatment with the Hsp90 inhibitor NVP-AUY922 in SKBR3 xenografts [53]. And Kramer-Marek et al. studied the changes of HER2 expression downregulated by Hsp90 inhibitor, 17-DMAG, through the affibody-based PET tracer N-[2-(4-[^18^F]fluorobenzamido)ethyl]maleimide (^18^F-FBEM)-Z_HER2:342_ [54]. The optical imaging probe, anti-Her2 Affibody-AlexaFluor680, was also reported to noninvasively monitor changes in HER2 expression *in vivo* as a response to Hsp90 inhibitor therapy with results similar to the imaging-based response measured by PET [55].

Techniques described above in PET can be adapted for human use and would allow noninvasive imaging of the pharmacodynamics of drug action which may lead to useful information for clinical trials in breast cancer therapy.

#### 3.2.2. EGFR

EGFR (HER1, ErbB1) has been found to be overexpressed in breast cancer. EGFR is a transmembrane protein which contributes to cell proliferation by binding to the epidermal growth factor (EGF) or the transforming growth factor alpha (TGF*α*). A variety of EGFR inhibitors have been developed based on the competition with EGF and TGF*α* [100]. Antibody-based, affibody-based, or EGF-based molecular probes for EGFR imaging of breast cancer have also been under active investigation. 

Modified tyrosine kinase inhibitor, [^11^C]-4-N-(3-bromoanilino)-6,7-dimethoxyquinazoline ([^11^C]PD153035), has been evaluated as a PET agent to measure EGFR expression in breast tumors. Wang et al. performed *ex vivo* biodistribution studies of [^11^C]PD153035 in nude mice bearing MDA-MB-468, A-549, and MDA-MB-231 xenografts [56]. This study showed that the uptake of [^11^C]PD153035 was correlated with EGFR expression in breast tumors. The PET radiotracer, ^11^C Iressa, has been reported in clinical trial for EGFR imaging in lung cancer, but no application in breast cancer imaging has been published.

A technique using streptavidin cadmium selenide/zinc sulfide quantum dots (Qdots) multiplexed with polyethylene glycol (PEG), epidermal growth factor (EGF), and ^99m^Tc-hydrazinonicotinamide was reported by Jung et al. [57]. Specific high-affinity EGFR targeting of ^99m^Tc-hydrazinonicotinamide EGF-PEG-Qdot was observed by confocal microscopy and SPECT imaging. Ke et al. reported a EGF-Cy5.5 fluorescent optical probe which imaged EGFR expression in breast cancer by NIR devices [58]. EGF-Cy5.5 accumulated only in EGFR-positive tumors, and the uptake was shown to be blocked by an anti-EGFR monoclonal antibody.

An anti-EGFR monoclonal antibody Erbitux-based NIR probe was used to image the level of EGFR expression *in vivo *[59]. Anti-EGFR antibody conjugated fluorescent nanoparticles (FNs probe) showed good sensitivity and exceptional photostability for breast cancer cell imaging. Z_EGFR:1907_, anti-EGFR affibody, modified with different NIR fluorescent dyes have been reported to specifically bind EGFR-positive breast cancer cells. Additional work showed fast tumor targeting ability and good tumor/tissue contrast as early as 0.5 hr after injection [60]. Alex680-Z_EGFR:1907_ and Cy5.5-Z_EGFR:1907_ displayed higher tumor/tissue ratios than those of the other two probes which made them better candidates as EGFR-targeted probes for optical imaging. These results indicate that optical imaging probes may be useful as EGFR-targeting contrast agent for noninvasive imaging of EGFR expression and monitoring of responses to molecularly targeted therapy. But their clinical application is limited due to poor light penetration through the body tissues and fluids.

#### 3.2.3. IGF-1R

Type 1 insulin-like growth factor receptor (IGF-1R) is a transmembrane tyrosine kinase receptor which plays a critical role in signaling cell survival and proliferation and has become a new target for breast cancer treatment [101]. IGF-1R-targeted therapy can be monitored by imaging of IGF-1R expression. Heskamp et al. radiolabeled R1507, a monoclonal antibody directed against the IGF-1R, with ^89^Zr and ^111^In for imaging of IFG-1R expression by PET and SPECT, respectively [61]. The radiolabeled tracers have been evaluated in a triple negative breast cancer mouse model. The upregulation of IGF-1R expression was also measured by SPECT with ^111^In labeled R1507 during 17*β*-estradiol treatment. Interestingly, tamoxifen treatment resulted in the downregulation of IGF-1R expression in MCF-7 xenografts. The study indicated that this technique can be used to monitor IGF-1R expression in breast cancer therapy and predict therapy response in individual patients [62]. AVE-1642, a humanised anti-IGF1R monoclonal antibody, was conjugated to the fluorophore, Alexa 680, and used to detect IGF-1R expression and monitor IGF-1R expression [63]. The results showed that AVE-1642-Alexa 680 selectively targeted IGF-1R which led to specific accumulation in xenograft tumors.

Peptide nucleic acids (PNAs) are artificially synthesized DNA/RNA in which the nucleobases are attached to a pseudopeptide backbone [102, 103]. PNAs are more stable against nuclease and protease hydrolysis. Coupling of PNA with IGF-1R targeting probe can increase its uptake of the breast cancer cells. Tian et al. reported SPECT imaging of breast cancer xenograft tumors with ^99m^Tc-peptide-PNA-peptide (^99m^Tc-WT4185) which is specific for both oncogene cyclin D1 (CCND1) and IGF1 receptor [64–66]. PNA was also conjugated to metal chelators and D(Cys-Ser-Lys-Cys), a cyclized peptide analogue of IGF-1, for scintigraphy, PET, and MRI [67, 68]. These probes were reported to enter breast cancer cells overexpressing IGF-1R and then hybridize specifically with CCND1 mRNA to produce strong xenograft tumor signals.

### 3.3. Imaging Probes on Breast Cancer Angiogenesis

Angiogenesis is the physiological process of forming new blood vessels from preexisting ones, a key requirement for tumor growth and metastasis. The biomarkers related to angiogenesis, including VEGF and its receptor (VEGF-R), integrins, fibronectin, and endostatin, were considered to be attractive targets for breast cancer imaging and therapy.

#### 3.3.1. VEGF Receptor

Vascular endothelial growth factor (VEGF) family consists of six groups: VEGF-A, VEGF-B, VEGF-C, VEGF-D, VEGF-E, and the placental growth factor (PIGF) [104]. There are three known receptors for the VEGF, known as VEGF receptor 1 (VEGFR1), receptor 2 (VEGFR2), and receptor 3 (VEGFR3). Among them, VEGFR2 is mainly expressed in endothelial cells and overexpressed in tumor neovasculature [105]. VEGFR2 and its downstream signaling factors have been used as potential therapeutic targets. A variety of VEGFR2 antagonists are now in clinical trials for the diagnosis and treatment of many solid tumors, including breast cancer. Currently, VEGFR2 targeted breast cancer imaging is under intense investigation. Wang et al. prepared a modified VEGF_121 _(VEGF(DEE)) which selectively bound VEGFR2 over VEGFR1. The resulting VEGFDEE was labeled with ^64^Cu via conjugation to DOTA for PET imaging to study breast tumor angiogenesis [69]. ^64^Cu-DOTA-VEGF(DEE) exhibited 20-fold higher binding affinity of VEGFR2 than that of VEGFR-1 in cell binding assays. MicroPET imaging studies showed that ^64^Cu-DOTA-VEGF(DEE) had comparable tumor targeting efficacy to ^64^Cu-DOTA-VEGF_121_ with reduced renal toxicity.

Lyshchik et al. reported a VEGFR-2 specific ultrasound contrast agent (UCAs) by conjugating microbubbles (MB) with biotinylated anti-VEGFR2 monoclonal antibodies [70]. The complex was used to investigate the expression of VEGFR2 on the vascular endothelium in 4T1 and 67NR breast cancer murine models. The study showed that the ultrasound signal intensities observed from the two-cell lines correlated with relative VEGFR2 expression in the two-tumor types suggesting that molecular ultrasonography could be a potential technique for the noninvasive investigation of tumor vasculature.

Levashova et al. developed a ^99m^Tc-labeled single-chain VEGF (scVEGF) to monitor breast cancer treatment with sunitinib, a small-molecule VEGFR inhibitor [71]. The SPECT imaging with this VEGF-based tracer showed decreased VEGFR expression in tumor endothelium during treatment.

#### 3.3.2. Integrin

**Figure 3:**
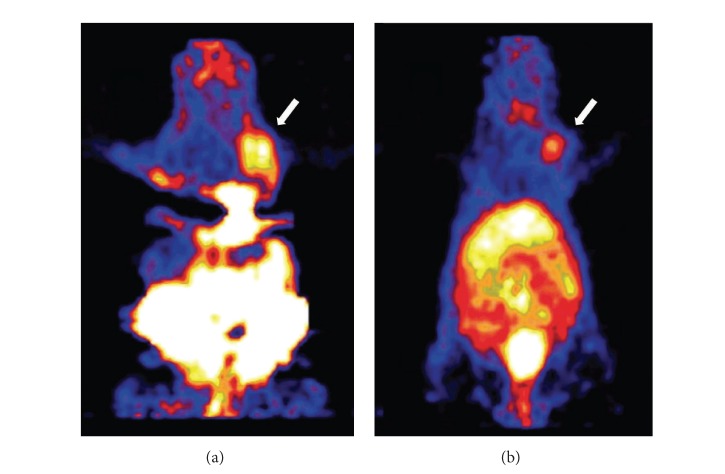
MicroPET imaging of orthotopic MDA-MB-435 breast cancer xenograft tumors in the right mammary fat pad (white arrow) following administration of 200 *μ*Ci of [^18^F]FB-RGD at 60 mins p.i. (a) and 400 *μ*Ci of ^64^Cu-DOTA-RGD at 2 hrs p.i. (b). Adapted from [72].

**Figure 4:**
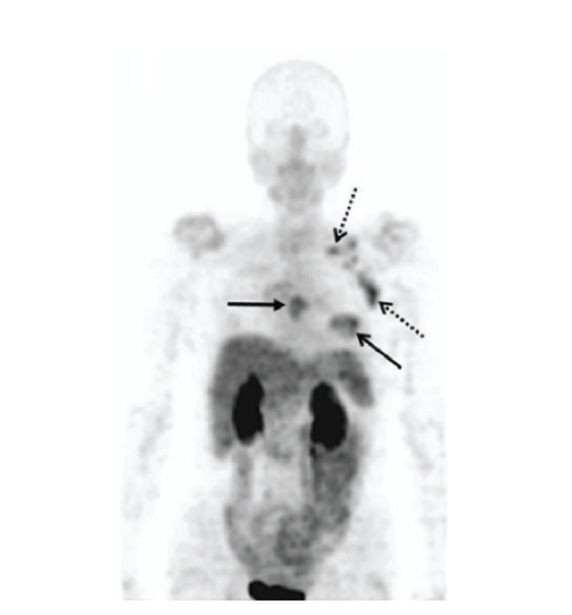
Maximum-intensity projection (MIP) of ^18^F-galacto-RGD PET in a patient with invasive ductal breast cancer of left breast (arrow, open tip), axillary and supraclavicular lymph-node metastases on left side (arrows, open tip, dotted line), and an osseous metastasis to the sternum (arrow, closed tip). Reprinted by permission of the Society of Nuclear Medicine from Beer et al. [75].

Integrins are cell adhesion receptors important for cell-extracellular matrix and cell-cell interactions. Among the many subtypes within this class, integrin *α*_*v*_ *β*_3_ has been shown to strongly correlate with tumor angiogenesis and metastasis. It has been demonstrated that integrin *α*_*v*_ *β*_3_ is overexpressed on both endothelial and tumor cells in breast cancer. Many integrin *α*_*v*_ *β*_3_-targeted imaging probes have been developed including high affinity arginine-glycine-aspartic acid (RGD) peptides. For example, ^64^Cu-DOTA-RGD was evaluated in murine orthotopic MDA-MB-435 human breast cancer model and compared with [^18^F]FB-RGD and ^125^I-RGD (Figure 3) [72]. The results indicated that all three radiotracers had fast blood clearance and high tumor/blood and tumor/muscle ratios. Although the ^64^Cu-DOTA-RGD and [^18^F]FB-RGD exhibited lower tumor uptake than ^125^I-RGD, likely due to a bulky 4-[^18^F]fluorobenzoyl group or ^64^Cu-DOTA complex, they are still suitable tracers for PET imaging of *α*_*v*_ *β*_3_ integrin expression in breast cancer. Moreover, the radiolabeled dimeric RGD peptides ^64^Cu-DOTA-E [c(RGDyK)] and ^64^Cu-DOTA-E [c(RGDfK)] were reported to have high and specific tumor uptake in a human breast cancer xenograft and showed better tumor retention than the corresponding monomeric RGD which may be due to increased binding affinity [73]. Cai et al. reported a series of ^18^F-labeled RGD peptides for PET imaging of integrin expression based on a new method of labeling RGD peptides through a thiol-reactive synthon, N-[2-(4-^18^F-fluorobenzamido)ethyl]maleimide (^18^F-FBEM) [74]. Both ^18^F-FBEM-SRGD (RGD monomer) and ^18^F-FBEM-SRGD2 (RGD dimer) had integrin-specific tumor uptake in subcutaneous orthotopic MDA-MB-435 xenografts. Beer et al. studied the tumor uptake patterns of the *α*_*v*_ *β*_3_-selective PET tracer ^18^F-galacto-RGD in sixteen patients with primary (*n* = 12) or metastatic breast cancer (*n* = 4) [75]. The results showed that all the primary tumor and metastasis were clearly identified although the standard uptake values were heterogeneous, suggesting varying levels of *α*_*v*_ *β*_3_ overexpression (Figure 4). Mühlhausen et al. reported ^68^Ga-DOTA-E-[c(RGDfK)] as a PET tracer suitable for monitoring bone metastases in a breast cancer mouse model [76].

Wu et al. reported the targeting and imaging of MDA-MB-231 human breast cancer cells using RGD peptide-labeled fluorescent silica nanoparticles (FSiNPs) as an optical imaging probe [77]. The contrast agent exhibited high target binding affinity to MDA-MB-231 breast cancer cells *in vitro* and good tumor uptake in breast cancer xenograft.

There are numerous RGD-based SPECT imaging probes reported in the literature. Zhang and Chen reported ^99m^Tc (I) tricarbonyl complex of cyclic RGD peptide for integrin *α*_*v*_ *β*_3_ receptor-targeted SPECT imaging [78]. ^99m^Tc-labeled cyclic RGD tetramer E[E[c(RGDfK)]_2_]_2_ and its 6-hydrazinonicotinamide conjugate (HYNIC-tetramer) was developed by Liu et al. [79]. Using xenograft models, the authors showed that the tetramer, E[E[c(RGDfK)]_2_]_2_, is a better integrin *α*_*v*_ *β*_3_-targeting agent than its monomeric and dimeric analogues with higher tumor uptake and tumor/blood ratio.

NC100692 is a cyclic, RGD-containing, and synthetic peptide with high affinity to integrins *α*_*v*_ *β*_3_ and *α*_*v*_ *β*_5_. In a proof-of-concept study performed by Bach-Gansmo et al., 19 of 22 malignant lesions were clearly detected by ^99m^Tc-NC100692 (86%) [80]. More recently, Axelsson et al. performed a phase 2a study in 10 patients with breast cancer by ^99m^Tc-NC100692 scintigraphy. One of seven metastases in liver, 4 of 5 in lung, 8 of 17 in bone, and 1 of 1 in the brain were detected after administration of ^99m^Tc-NC100692 [81].

For MRI imaging, N-(2-hydroxypropyl)methacrylamide (HPMA) copolymer-gadolinium(Gd)-RGDfK conjugates were developed as a contrast agent to detect integrin *α*_*v*_ *β*_3_ in breast cancer [82]. Ultrasonography using microbubbles (MBs) coupled with RGD peptide as contrast agents was also applied for integrin-targeted breast tumor imaging [5].

## 4. Conclusion

The goal of molecular imaging is not only to detect and stage disease but also to determine predictors of response or resistance to available therapies. Due to the fact that each breast tumor and its host environment may have unique features, optimal treatment and expected response for individual patient may not necessarily be universal. Molecular imaging techniques could play an important role for targeted therapy evaluation of breast cancer. With the introduction of breast tumor-specific imaging probes, it provides evidence of the on-target drug effect noninvasively and has great potential to predict which patient will benefit most from specific drugs or interventions and to rapidly monitor efficacy. Currently ^18^F-FDG is still the most extensively clinical used molecular imaging probe in breast cancer. Targeted ER and HER2 imaging is under intense preclinical and clinical investigation; however, these agents would apply only to ER or HER2-positive subtypes. Imaging probes targeting other major cellular receptors or biomarkers, such as VEGF and EGFR, are in demand for breast cancer diagnosis and therapy evaluation especially for triple-negative subtype patients. Hopefully as the fundamental molecular mechanisms of breast cancer are better understood, new targets will be elucidated which can lead to the development of next generation pathway-specific diagnostic agents. Such new agents will allow us to visualize changes in breast cancer at the molecular and cellular levels to fulfill the goals of early detection, characterization, and personalized therapy for breast cancer patients.

## Figures and Tables

**Table 1:** Selected molecular imaging probes for breast cancer.

Receptor/biomarker	Imaging probe	Imaging modality	In clinic/clinical trial	Reference
Glucose metabolism	^ 18^F-FDG	PET	*√*	[9–16]

DNA synthesis	^ 18^F-FLT	PET	*√*	[17–22]

ER	^ 18^F-FES and its analogs	PET	*√*	[23–25]
	Z-[^123^I]MIVE	Gamma imaging	*√*	[26, 27]
	^ 99m^Tc(I)-Estradiol-pyridin-2-yl hydrazine derivatives	SPECT		[28]
	^ 99m^Tc-Glutamate peptide estradiol (GAP-EDL)	SPECT		[29]
	^ 18^F-Fluorotamoxifen	PET	*√*	[30, 31]
	^ 18^F-Labeled cyclofenil analogues	PET		[32, 33]
	^ 99m^Tc-DTPA-TOR	SPECT		[34]
	^ 11^C-Labeled tetrahydroisoquinoline derivatives	PET		[35]
	EPTA-Gd/TPTA-Gd	MRI		[4]

PR	[^18^F]FMNP	PET		[36]
	[^18^F]FENP	PET	*√*	[37]
	[^18^F]FPTP	PET		[38, 39]

HER2	^ 89^Zr-Labeled trastuzumab	PET	*√*	[40]
	^ 111^In-Labeled trastuzumab	SPECT	*√*	[41]
	^ 111^In-Labeled pertuzumab	SPECT		[42]
	^ 99m^Tc-Labeled 2Rs15d	SPECT		[43]
	Herceptin-nanoparticles	MRI		[44]
	PAION-Ab	MRI		[45]
	^ 111^In-ICG-panitumumab/^111^In-ICG-trastuzumab	SPECT/optical imaging		[46]
	^ 68^Ga-ABY-002/^111^In-ABY-002	SPECT		[47]
	^ 99m^Tc-Z_HER2:2395_-Cys	SPECT		[48]
	Streptavidin-functionalized SPIO and biotinylated HER2-specific affibody	MRI		[49]
	Affibody-based fluorescence agent	Optical imaging		[50, 51]

HSP90 therapy response	^ 111^In-, ^64^Cu-, and ^68^Ga-labeled DOTA-conjugated Herceptin fragment	PET		[52]
	^ 89^Zr-labeled trastuzumab	PET		[53]
	(^18^F-FBEM)-Z_HER2:342_	PET		[54]
	Anti-Her2 Affibody-AlexaFluor680	Optical imaging		[55]

EGFR	[^11^C]PD153035	PET		[56]
	^ 99m^Tc-Hydrazinonicotinamide EGF-PEG-Qdot	Confocal microscopy		[57]
	EGF-Cy5.5	Optical imaging		[58]
	Anti-EGFR antibody conjugated FNs	Optical imaging		[59]
	Alex680-Z_EGFR:1907_ and Cy5.5-Z_EGFR:1907_	Optical imaging		[60]

IGF-1R	^ 89^Zr or ^111^In labeled R1507	SPECT or PET		[61, 62]
	AVE-1642-Conjugated Alexa 680	Optical imaging		[63]
	^ 99m^Tc-Peptide-PNA-peptide	SPECT		[64–66]
	Metal-chelator-PNA-peptides	Scintigraphy, PET, or MRI		[67, 68]

	^ 64^Cu-DOTA-VEGF(DEE)	PET		[69]
VEGFR	Anti-VEGFR2 Monoclonal antibody-conjugated UCA	Ultrasonography		[70]
	^ 99m^Tc-labeled single-chain VEGF	SPECT		[71]

Integrin	^ 64^Cu-DOTA-RGD, [^18^F]FB-RGD, and ^125^I-RGD	PET		[72]
	^ 64^Cu-DOTA-dimer RGD	PET		[73]
	^ 18^F-FBEM-SRGD (RGD monomer) and ^18^F-FBEM-SRGD2 (RGD dimer)	PET		[74]
	^ 18^F-galacto-RGD	PET	*√*	[75]
	^ 68^Ga-DOTA-E-[c(RGDfK)]	PET		[76]
	RGD peptide-labeled FSiNPs	Optical imaging		[77]
	^ 99m^Tc(I) Tricarbonyl complex of cyclic RGD peptide	SPECT		[78]
	^ 99m^Tc-labeled cyclic RGD tetramer	SPECT		[79]
	^ 99m^Tc-NC100692	Scintigraphy	*√*	[80, 81]
	HPMA copolymer-Gd-RGDfK	MRI		[82]
	MBs-RGD	Ultrasonography		[5]

MRI: dynamic contrast-enhanced magnetic resonance imaging; PET: positron emission tomography; SPECT: single-photon emission computed tomography; FDG: ^18^F-fluorodeoxyglucose; FLT: ^18^F-fluorothymidine; ER: estrogen receptor; FES: 16*α*-[^18^F]-fluoro-17*β*-estradiol; Z-[^123^I]MIVE, ^123^Iodine labelled cis-11*β*-methoxy-17*α*-iodovinyloestradiol; EPTA-Gd/TPTA-Gd: pyridine-tetra-acetate-Gd(III) chelate (PTA-Gd) conjugated to 17*β*-estradiol/tamoxifen; PR: progesterone receptor; [^18^F]FMNP: 21-[^18^F]fluoro-16-*α*-methyl-19-norprogesterone; [^18^F]FENP: 21-[^18^F]-Fluoro-16*α*-ethyl-norprogesterone; [^18^F]-FPTP: 4-[^18^F]fluoropropyl-Tanaproget; HER2: human epidermal growth factor receptor 2; PAION-Ab: poly(amino acid) coated iron oxide nanoparticles conjugated with HER2 antibody; SPIO: superparamagnetic iron oxide; HSP90: heat shock protein 90; ^18^F-FBEM: N-[2-(4-[^18^F]fluorobenzamido)ethyl]maleimide; EGFR: epidermal growth factor receptor; IGF-1R: type 1 insulin-like growth factor receptor; VEGFR: vascular endothelial growth factor receptor; UCA: ultrasound contrast agents; RGD: arginine-glycine-aspartic acid peptide; FSiNPs: fluorescent silica nanoparticles; HPMA: N-(2-hydroxypropyl)methacrylamide; MBs: microbubbles.
